# HLA-G Polymorphism (rs16375) and Acute Rejection in Liver Transplant Recipients

**DOI:** 10.1155/2014/814182

**Published:** 2014-01-23

**Authors:** Negar Azarpira, Mahdokht H. Aghdaie, Kurosh Kazemi, Bita Geramizadeh, Masumeh Darai

**Affiliations:** ^1^Organ Transplant Research Center, Namazi Hospital, Shiraz University of Medical Sciences, Zand Street, Shiraz 7193711351, Iran; ^2^Transplant Center, Shiraz University of Medical Sciences, Shiraz 7193711351, Iran

## Abstract

*Background*. HLA-G molecules exhibit immunomodulatory properties that can delay graft rejection. The 14 bp insertion/deletion polymorphism (INDEL) (rs16375) influences the stability of final HLA-G mRNA and its soluble isoforms. *Objective*. The present study aimed to investigate the possible association between this polymorphism and the incidence of acute rejection in Iranian liver transplant recipients. *Methods*. Different genotypes were evaluated by PCR. The patients who had acute rejection within 6 months after transplantation were classified as acute rejection (AR) group, while others were considered as nonacute rejection (NAR) group. *Results*. Among the recipients, 21 patients (21%) had at least one episode of AR, while the other 79 patients (79%) had normal liver function. No significant differences were found between the two groups regarding sex, MELD score, and primary liver disease. Also, no difference was observed concerning rs16375 genotype and allele frequency (*P* = 0.44, OR: 0.69; CI: 0.21–2.10). *Conclusion*. The study results revealed no significant difference between the AR and the NAR groups regarding the 14 bp INDEL genotypes and alleles. Further studies are recommended to be conducted on other polymorphic sites as well as monitoring of serum HLA-G concentration in order to ascertain the potential implications of this marker in our population.

## 1. Introduction

The human leukocyte antigen-G (HLA-G) belongs to the major histocompatibility complex (MHC) located on the short arm of chromosome 6. During HLA-G gene transcription, 7 different isoforms are produced, four of which are membrane bound (HLA-G1 through HLA-G4) and three are soluble (HLA-G5 through HLA-G7) [1–3].

HLA-G is predominantly expressed at the maternal-fetal interface and plays an important role in fetal-maternal tolerance which is the perfect example of a semiallograft [4].

HLA-G protects the fetus against the immunological damage caused by maternal natural killer and T-cytotoxic cells (CTLs) during pregnancy [5].

In nonpathological conditions, expression of HLA-G is detected in thymus, cornea, proximal nail matrix, pancreas, and hematopoietic stem cells. There is strong evidence that HLA-G expression is associated with the reduced incidence of acute and chronic rejection in solid organ transplantation [2, 3].

HLA-G is involved in graft acceptance following human allotransplantation, such as heart, lung, liver, and kidney [6–8]. The endomyocardial cells in the heart, biliary epithelial cells of the liver, and the tubular epithelial cells in the kidney are the important targets of the immune system in transplant rejection. A positive correlation has been found between detection of HLA-G in both serum and tissue biopsies and lower incidence of acute rejection episodes [6–8]. Therefore, HLA-G is considered as a tolerogenic marker in transplanted recipients.

Mesenchymal stem cells also express the HLA-G. These cells are used in bone marrow and solid organ transplantation and are considered as an endogenous source of HLA-G with tolerogenic properties [9]. In animal models with allogeneic skin transplants, a single injection of exogenous HLA-G-coated microbeads significantly prolonged the transplant tolerance [10].

Castelli et al. believed that, in contrast to the classical HLA class I loci, HLA-G coding region has limited variability in worldwide population studies [11]. The variation is mainly located in 5′URR and 3′UTR [12]. Many SNPs have been located in the HLA-G promoter region (Berger et al. 2010), which may affect the expression of HLA-G [5].

The polymorphic sites at 5′URR have linkage disequilibrium (LD) with those identified at 3′UTR [4, 12].

Overall, three well-known polymorphic sites, which are associated with the regulation of HLA-G expression levels, have been identified at 3′UTR. The 14 bp deletion/insertion polymorphism influences the stability of final mRNA [4, 12]. The HLA-G alleles presenting the 14 bp (5′-ATTTGTTCATGCCT-3′) sequence have been associated with lower mRNA production for most membrane and soluble isoforms [2, 13].

The presence of guanine in the position 3142 which increases the affinity of specific microRNAs (miR-148a, miR-148b, and miR-152) for HLA-G mRNA decreases HLA-G expression [14]. In addition, the presence of an adenine at position 3187 modifies an AU-rich motif in the mRNA and decreases its stability [15].

There is a strong LD among these three major 3′UTR polymorphic sites. The 14 bp insertion is always associated with 3142G and 3187A alleles [4, 12, 16].

As the expression of sHLA-G may be influenced by genetic variants of the *HLA-G* gene and the 14 bp insertion/deletion is a functional polymorphism, the possible association between this genetic variant and graft acceptance was investigated [16, 17].

Crispim et al. investigated relationship between the 14 bp ins./del. polymorphism of the HLA-G gene and kidney allograft outcome. They found no significant difference between the control and renal transplant patients regarding allelic frequencies of the 14 bp ins./del. polymorphism [18]. This result was also confirmed by another study which was conducted on renal transplant recipients [19]. The present study aims to investigate the possible association between 14 bp ins./del. HLA-G gene and the incidence of acute rejection (AR) in liver transplant recipients.

## 2. Materials and Methods

### 2.1. Study Population

This study was conducted on 100 Iranian liver transplant recipients with a regular followup between June 2011 and March 2012. The Ethics Committee of Shiraz University of Medical Sciences approved the protocol, and written informed consents were obtained from all the participants. The study was conducted according to the Declaration of Helsinki. The patients were followed up for 6 months and episodes of AR were recorded in this period. The patients' characteristics and detailed medical history were derived from an electronic database that is maintained in our department.

### 2.2. Immunosuppressant Regimen and Rejection Diagnosis

The routine immunosuppressant regimen consisted of 1-2 mg/day tacrolimus, 500 mg/day mycophenolate mofetil, and 120 mg prednisone q12 h. The drugs were administered orally. AR diagnosis was based on the clinical parameters (fever and elevation of bilirubin and/or transaminase levels in the absence of vascular problems or biliary obstruction) or biopsy findings according to Banff criteria [20].

The patients with AR episode received high doses (500 mg) of methylprednisolone for 3 consecutive days. Overall, the patients who had AR within 6 months after the transplantation were classified as the AR group, while the other patients were considered as the nonacute rejection (NAR) group.

### 2.3. Genotyping

Genomic DNAs were extracted from peripheral white blood cells using the commercial extraction kit (DNG plus DNA Extraction Kit, Cinagene Company, Tehran, Iran). Moreover, HLA-G 14 bp insertion/deletion polymorphism (rs16375) was amplified by polymerase chain reaction (PCR) [21] with the forward primer HLAG1 5′-GTGATGGGCTGTTTAAAGTGTCACC-3′ and the reverse primer HLAG2 5′-GGAAGGAATGCAGTTCAGCATGA-3′.

Fragments of 224 or 210 bp were obtained depending on the absence or presence of the 14 bp deletion. PCR was carried out under the following conditions: a denaturation step at 92°C for 5 minutes and 30 cycles at 92°C for 30 seconds, 64°C for 1 minute, and 72°C for 2 minutes. The PCR products were analyzed by electrophoresis in 4% agarose gel stained with ethidium bromide.

### 2.4. Statistical Analyses

All the statistical analyses were performed using the SPSS software package, version 16.0 (SPSS Inc., Chicago, IL, USA), and Epi info 2000. Continuous variables were compared using *t*-test.

Besides, between-group differences in the clinical findings and the frequency of alleles and genotypes in the patients with or without AR episode were compared using Pearson's *χ* ^2^ test or Fisher's exact test. *P* value <0.05 was considered to be statistically significant.

Odds ratios (ORs) and 95% confidence intervals (CIs) were calculated to estimate the risk for the incidence of rejection.

## 3. Results

This study was conducted on 100 patients. Among the study patients, 55 were male and 45 were female, with the mean age of 22.6 ± 12.9 years. In addition, 21 recipients (21%) were in the AR group, while 79 ones (79%) were in the NAR group. The main causes for liver transplantation included chronic hepatitis B, cryptogenic cirrhosis, primary sclerosing cholangitis, and autoimmune hepatitis (Table 1). The results revealed no significant differences between the two groups regarding sex, model for end-stage liver disease (MELD) score [17], and primary liver disease (Table 1).

There was statistically significant difference between AR and NAR when considering the kind of donor (living and deceased) (*P* = 0.01).

The genotype determined at the position rs16375 was as follows: homozygous −14/−14 bp genotype presented in 35 patients, heterozygous −14/+14 bp genotype presented in 50 patients, and homozygous +14/+14 bp genotype presented in 15 patients (Table 2).

No significant difference was found between the AR and NAR groups regarding rs16375 genotype and allele frequency (Table 2).

## 4. Discussion

HLA-G is a nonclassic class I HLA that affects the immune system by suppressing the cytotoxic effect of CD8^+^ T-cell/NK cells, inhibits dendritic cell function, decreases the proliferation of CD4^+^ T-cell, and promotes T helper 2 (Th2) type responses [22–25]. The tolerogenic HLA-G was carried out by interaction of HLA-G with its receptor ILT2. This affected the cyclins and inhibitory kinases and finally induced a cell-cycle arrest at the G1 phase [26].

The association between the involvement of HLA-G soluble molecules and the 14 bp ins./del. polymorphism in the HLA-G gene and occurrence of graft-versus-host disease has been studied in bone marrow transplantation and other complications in solid organ transplantation [27]. Jin et al. measured the level of soluble human leukocyte antigen (sHLA-G) in renal transplant patients [17]. They found that the patients with high sHLA-G levels experienced lower rejection episodes. Therefore, they suggested that HLA-G was involved in induction of immunologic tolerance in renal transplant recipients [17]. Increased soluble level of HLA-G with expression of HLA-G in endomyocardial biopsy was reported to be associated with lower incidence of rejection episodes in heart transplant recipients [6, 28, 29].

In the current study, we investigated the possible impact of 14 bp ins./del. polymorphism in the HLA-G gene on the graft outcome in 100 Iranian patients undergoing allogenic liver transplantation.

We found statistically significant difference between AR and NAR when considering the kind of donor (living and deceased). In AR group, about 60% of the recipients received organ from deceased donor. Berg et al., conducted a study on liver transplant recipients who received organ from living or deceased donor and compared the outcome and survival rate [30]. Their results showed that receipts of a living donor liver transplant were associated with improved survival compared with continued waiting for a deceased donor liver transplant [30].

Our results revealed no association between the 14 bp polymorphism and AR as a posttransplant complication. Moreover, no statistically significant differences were found between the groups regarding the distribution of 14 bp ins./del. alleles. Waterhouse conducted a study on allogeneic hematopoietic cell transplant recipients. Their results showed no association between the HLA-G 14 bp polymorphism, the soluble HLA-G level, and acute graft-versus-host disease or death [27]. However, Liu et al. revealed that the increased level of soluble HLA-G5 in allogeneic hematopoietic stem cell transplant recipients might be a predictor of the occurrence of graft-versus-host disease. Therefore, the HLA-G5 was suggested as an individual surrogate marker for prophylaxis against a GVHD [3].

Furthermore, Aghdaie et al. investigated the 14 bp ins./del. polymorphism in renal transplant recipients and reported no significant association between AR episodes and different genotypes or alleles [21]. Also, Crispim et al. studied the kidney transplant patients and showed no significant difference between the control and kidney transplant patients regarding the allelic frequencies of the 14 bp ins./del. polymorphism [18].

Littera et al. studied the HLA-G 14 bp insertion/deletion polymorphism as a genetic risk marker for acute and/or chronic deterioration in kidney transplant recipients. They observed a significantly higher incidence of chronic renal dysfunction with allograft loss in the recipients homozygous for the HLA-G 14 bp deletion polymorphism [1].

Jin et al. showed an increased frequency of homozygous genotype +14/+14 bp in renal transplant patients with acute rejection in comparison to the patients without normal kidney function [19].

Zarkhin et al. monitored the serum levels of HLA-G1 and HLA-G5 in pediatric and adult liver transplant recipients. Their result confirmed that the increased serum level of HLA-G was associated with operational tolerance and more favorable outcomes in hepatic transplant patients [8].

In another study which was conducted in Turkey, Baştürk et al. found a correlation between higher HLA-G serum levels and better liver function tests, such as aspartate aminotransferase, alanine aminotransferase, direct bilirubin, total bilirubin, and alkaline phosphatase [31].

Créput et al. investigated the expression of HLA-G in kidney and liver biopsies of combined double organ transplanted patients. They found a significant association between HLA-G expression in liver biliary epithelial cells and the absence of liver rejection. No acute or chronic rejection of the kidney graft was observed in the patients in whom HLA-G was expressed in the biliary epithelial cells of the liver graft. The biliary epithelial cells might express both the membrane bound and the soluble form of HLA-G molecules [32].

Membrane-bound isoforms has a local immunomodulatory role, while the soluble molecules have systemic inhibitory properties especially on NK and T cells which are the major cells involved in graft rejection. Therefore, they suggested that HLA-G as a tolerogenic antigen was produced by the liver allograft and might be involved in the acceptance of simultaneously transplanted organs [32].

In another study on dual liver-kidney transplanted recipients, Creput et al. detected higher concentrations of serum HLA-G with lower incidence of acute rejection in double organ transplanted patients but not in kidney transplanted recipients. They suggested that serum HLA-G could be used for monitoring of graft acceptance and adjustment of immunosuppressive drugs [33].

Overall, the mentioned studies confirmed that higher serum HLA-G levels were associated with lesser solid organ transplant reelection risk [32–35]. However, with respect to the insertion/deletion polymorphism (INDELs), the results are controversial.

To the best of our knowledge, no published article has evaluated the possible association between HLA-G genetic polymorphism and liver transplant outcome. The findings of the present study indicated no significant association between HLA-G 14 bp INDEL polymorphism and acute rejection after liver transplantation. Although the 14 bp INDEL polymorphism is responsible for the posttranscriptional regulation of the HLA-G gene, further studies are recommended to be conducted on other polymorphic sites which are located at 5′URR that is close to the transcription factor binding sites or at the 3′UTR that influences HLA-G mRNA availability.

Moreover, since liver is considered as a de novo site for HLA-G synthesis, genotyping of 14 bp INDEL is suggested in liver donors. Besides, monitoring of the patient with serum HLA-G level as well as tissue expression in liver biopsies may anticipate the rejection-free survival of the liver grafts.

## Figures and Tables

**Table 1:** Demographics of liver graft recipients.

Parameter	ARs (%)	NARs (%)
Number of patients	21 (21)	79 (79)
Recipient gender		
Male	13 (68)	32 (61)
Female	8 (32)	47 (39)
Cold ischemic time (hour)	11.2 ± 3.4	10.3 ± 1.2
Recipient age (years, mean ± SD)	31.45 ± 13.3	32.1 ± 10.1
Donor age (years, mean ± SD)	33.9 ± 11.3	34.44 ± 12.1
Donor gender		
Male	12 (58)	49 (62)
Female	9 (42)	30 (38)
Primary disease		
Hepatitis B (28)	6 (30)	22 (27)
Cryptogenic (24)	4 (21)	20 (26)
Primary sclerosing cholangitis (13)	5 (24)	8 (10)
Autoimmune hepatitis (11)	3 (15)	8 (10)
Biliary atresia (6)	0	6 (8)
Hypercholesteremia (5)	0	5 (7)
Wilson disease (3)	0	3 (4)
Tyrosinemia (2)	0	2 (2)
Alcoholism (2)	0	2 (2)
Crigler Najjar syndrome (2)	0	2 (2)
Hepatocellular carcinoma (2)	1 (5)	1 (1)
Hepatitis C virus (1)	0	1 (1)
Fulminate hepatitis (1)	1 (5)	0
Living donor	8 (38)	10 (13)
Deceased donor	13 (62)	69 (87)
Histological grade of rejection		
I	13 (61)	
II	5 (24)	
III	3 (15)	

ARs: acute rejection; NARs: nonacute rejection.

**Table 2:** Distribution of HLA-G 14 bp insertion/deletion (rs16375) genotype/allele in acute rejection (AR) and nonacute rejection (NAR) groups.

HLA-G genotype	ARs (%)	NARs (%)	*P* value	OR (95% CI)
−14/−14	6 (29)	29 (37)	0.48	0.69 (0.21–2.18)
−14/+14	13 (62)	37 (47)	0.21	1.84 (0.62–5.54)
+14/+14	2 (9)	13 (16)	0.34	0.53 (0.08–2.85)

−14/−14	6 (29)	29 (37)	0.48	0.69 (0.21–2.10)
−14/+14 and +14/+14	15 (71)	50 (63)		

HLA-G allele				
−14 insertion	25 (60)	95 (60)	0.94	0.98 (0.46–2.07)
+14 deletion	17 (40)	63 (40)		

## References

[1] Littera R, Piredda G, Pani A (2013). Role of human leukocyte antigen-G 14-base pair polymorphism in kidney transplantation outcomes. *Journal of Nephrology*.

[2] Hviid TVF, Hylenius S, Rørbye C, Nielsen LG (2003). HLA-G allelic variants are associated with differences in the HLA-G mRNA isoform profile and HLA-G mRNA levels. *Immunogenetics*.

[3] Liu H, Chen Y, Xuan L (2013). Soluble human lukocyte antigen G molecule expression in allogeneic hematopoietic stem cell transplantation: good predictor of acute Graft-versus-Host disease. *Acta Haematologica*.

[4] Castelli EC, Mendes-Junior CT, Veiga-Castelli LC, Roger M, Moreau P, Donadi EA (2011). A comprehensive study of polymorphic sites along the HLA-G gene: implication for gene regulation and evolution. *Molecular Biology and Evolution*.

[5] Berger DS, Hogge WA, Barmada MM, Ferrell RE (2010). Comprehensive analysis of HLA-G: implications for recurrent spontaneous abortion. *Reproductive Sciences*.

[6] Sheshgiri R, Rouas-Freiss N, Rao V (2008). Myocardial HLA-G reliably indicates a low risk of acute cellular rejection in heart transplant recipients. *Journal of Heart and Lung Transplantation*.

[7] Rebmann V, Bartsch D, Wunsch A (2009). Soluble total human leukocyte antigen class I and human leukocyte antigen-G molecules in kidney and kidney/pancreas transplantation. *Human Immunology*.

[8] Zarkhin V, Talisetti A, Li L (2010). Expression of soluble HLA-G identifies favorable outcomes in liver transplant recipients. *Transplantation*.

[9] Naji A, Rouas-Freiss N, Durrbach A, Carosella ED, Sensébé L, Deschaseaux F (2013). Concise review: combining human leukocyte antigen G and mesenchymal stem cells for immunosuppressant biotherapy. *Stem Cells*.

[10] Urosevic M (2007). HLA-G in the skin—friend or foe?. *Seminars in Cancer Biology*.

[11] Castelli EC, Mendes CT, Donadi EA (2007). HLA-G alleles and HLA-G 14 bp polymorphisms in a Brazilian population. *Tissue Antigens*.

[12] Castelli EC, Mendes-Junior CT, Deghaide NHS (2010). The genetic structure of 3′untranslated region of the HLA-G gene: polymorphisms and haplotypes. *Genes and Immunity*.

[13] Hviid TVF, Rizzo R, Melchiorri L, Stignani M, Baricordi OR (2006). Polymorphism in the 5′ upstream regulatory and 3′ untranslated regions of the HLA-G gene in relation to soluble HLA-G and IL-10 expression. *Human Immunology*.

[14] Veit TD, Chies JAB (2009). Tolerance versus immune response—microRNAs as important elements in the regulation of the HLA-G gene expression. *Transplant Immunology*.

[15] Yie S-M, Li L-H, Xiao R, Librach CL (2008). A single base-pair mutation in the 3′-untranslated region of HLA-G mRNA is associated with pre-eclampsia. *Molecular Human Reproduction*.

[16] Larsen MH, Hviid TVF (2009). Human leukocyte antigen-G polymorphism in relation to expression, function, and disease. *Human Immunology*.

[17] Jin HL, Li CR, Xiao L (2012). Clinical relevance of sHLA-G-mediated with better graft acceptance in early posttransplantation. *Transplantation Proceedings*.

[18] Crispim JCO, Mendes-Junior CT, Wastowski IJ (2008). Frequency of insertion/deletion polymorphism in exon 8 of HLA-G and kidney allograft outcome. *Tissue Antigens*.

[19] Jin ZK, Xu CX, Tian PX (2012). Impact of HLA-G 14-bp polymorphism on acute rejection and cytomegalovirus infection in kidney transplant recipients from northwestern China. *Transplant Immunology*.

[20] Demetris A, Adams D, Bellamy C (2000). Update of the international Banff schema for liver allograft rejection: working recommendations for the histopathologic staging and reporting of chronic rejection. *Hepatology*.

[21] Aghdaie MH, Azarpira N, Kazemi K, Geramizadeh B, Darai M, Malekhoseini SA (2011). Frequency of HLA-G exon 8 polymorphisms and kidney allograft outcome in Iranian population. *Molecular Biology Reports*.

[22] Bainbridge DRJ, Ellis SA, Sargent IL (2000). HLA-G suppresses proliferation of CD4^+^ T-lymphocytes. *Journal of Reproductive Immunology*.

[23] Tripathi P, Abbas A, Naik S, Agrawal S (2004). Role of 14-bp deletion in the HLA-G gene in the maintenance of pregnancy. *Tissue Antigens*.

[24] Selmani Z, Naji A, Zidi I (2008). Human leukocyte antigen-G5 secretion by human mesenchymal stem cells is required to suppress T lymphocyte and natural killer function and to induce CD4^+^CD25^high^FOXP3^+^ regulatory T cells. *Stem Cells*.

[25] Liang S, Baibakov B, Horuzsko A (2002). HLA-G inhibits the functions of murine dendritic cells via the PIR-B immune inhibitory receptor. *European Journal of Immunology*.

[26] Rouas-Freiss N, Paul P, Dausset J, Carosella ED (2000). HLA-G promotes immune tolerance. *Journal of Biological Regulators and Homeostatic Agents*.

[27] Waterhouse M (2013). Soluble HLA-G molecules and HLA-G 14-base pair polymorphism after allogeneic hematopoietic cell transplantation. *Transplantation Proceedings*.

[28] Lila N, Carpentier A, Amrein C, Khalil-Daher I, Dausset J, Carosella ED (2000). Implication of HLA-G molecule in heart-graft acceptance. *The Lancet*.

[29] Torres MI, Luque J, Lorite P (2009). 14-Base pair polymorphism of human leukocyte antigen-G as genetic determinant in heart transplantation and cyclosporine therapy monitoring. *Human Immunology*.

[30] Berg CL, Merion RM, Shearon TH (2011). Liver transplant recipient survival benefit with living donation in the model for endstage liver disease allocation era. *Hepatology*.

[31] Baştürk B, Karakayali F, Emiroğlu R (2006). Human leukocyte antigen-G, a new parameter in the follow-up of liver transplantation. *Transplantation Proceedings*.

[32] Créput C, Durrbach A, Menier C (2003). Human leukocyte antigen-G (HLA-G) expression in biliary epithelial cells is associated with allograft acceptance in liver-kidney transplantation. *Journal of Hepatology*.

[33] Creput C, Le Friec G, Bahri R (2003). Detection of HLA-G in serum and graft biopsy associated with fewer acute rejections following combined liver-kidney transplantation: possible implications for monitoring patients. *Human Immunology*.

[34] Rouas-Freiss N, LeMaoult J, Moreau P, Dausset J, Carosella ED (2003). HLA-G in transplantation: a relevant molecule for inhibition of graft rejection?. *American Journal of Transplantation*.

[35] Lemaoult J, Daouya M, Wu J, Loustau M, Horuzsko A, Carosella ED (2013). Synthetic HLA-G proteins for therapeutic use in transplantation. *FASEB Journal*.

